# Assessment of a proposed BMI formula in predicting body fat percentage among Filipino young adults

**DOI:** 10.1038/s41598-020-79041-3

**Published:** 2020-12-15

**Authors:** Michael Van Haute, Emer Rondilla, Jasmine Lorraine Vitug, Kristelle Diane Batin, Romaia Elaiza Abrugar, Francis Quitoriano, Kryzia Dela Merced, Trizha Maaño, Jojomaku Higa, Jianna Gayle Almoro, Darlene Ternida, J. T. Cabrera

**Affiliations:** 1 College of Medicine, De La Salle Medical and Health Sciences Institute, Dasmariñas City, Cavite Philippines; 2 College of Medicine, San Beda University, Mendiola, Manila Philippines

**Keywords:** Obesity, Obesity, Obesity

## Abstract

Body mass index (BMI), while routinely used in evaluating adiposity, cannot distinguish between fat and lean mass, and thus can misclassify weight status particularly among athletic, physically active, and tall- and short-statured individuals, whose lean-to-fat ratios and body proportions vary considerably from average individuals. Believing that the traditional BMI formula divides weight by too much with short people and by too little with tall people, University of Oxford professor L. N. Trefethen proposed a modified formula in computing BMI. This study was conducted among a sample of Filipino young adults (n = 190) to assess the performance of the modified BMI formula against the traditional one in: (1) predicting body fat percentage (%BF) measured using bioelectric impedance analysis, and (2) diagnosing overweight/obesity. Using robust polynomial regression analysis (covariates: age, waist circumference, smoking history and alcohol intake), the BMI quadratic models had the highest adjusted *R*^2^ and the lowest AIC and BIC for both sexes compared to the linear models. The AuROCs of the traditional BMI were higher than those of the proposed BMI, albeit nonsignificant. In conclusion, both traditional and modified BMIs significantly predicted %BF, as well as adequately discriminated between %BF-defined normal and overweight-obese states using optimal BMI cutoff values.

## Introduction

Obesity, described as abnormal or excessive fat accumulation, has been steadily growing in prevalence since the 1970s and has more than tripled over a forty-year period^1,2^. Relative to many high-income countries, the increase in obesity rates appears to be faster in Asia^3^. As of 2014, about 5.1% of the adult Filipino population was classified as obese, representing a 24% relative increase in the number of obese Filipino adults from 2010^4^. In this same 2014 report, the estimated proportion of overweight Filipino adults was 23.6%. While these figures are relatively low compared to the neighboring countries, they still translate to roughly 18 million obese and overweight individuals. This increasing trend in obesity is concerning because it imposes an additional burden on top of already-existing problems of undernutrition and infectious diseases typical in low- and middle-income countries^5^. In 2016, Philippine healthcare spending on obesity alone has already amounted to somewhere between US$ 500 million and US$ 1 billion^6^.

Currently, body mass index (BMI) is an accepted anthropometric measure used in screening for overweight and obesity, and for categorising individuals into different weight groups (underweight, normal, overweight, and obese). This is invariably due to its noninvasiveness and satisfactory correlation with body fat percentage (%BF)^7^. BMI is, however, limited in that it cannot distinguish between fat and lean body mass^8^, and is influenced by factors independent of height and weight, like age, sex, ethnicity, muscle mass, and activity level. As a result, its use can potentially misclassify the weight status of athletic, physically active, and tall- and short-statured individuals, whose lean-to-fat ratios and body proportions can vary considerably from average individuals. This BMI–body fat discordance carries a consequence of introducing important bias when estimating the effects related to obesity^9^, as well as the possibility of failing to identify individuals at risk for chronic diseases^10,11^.

Although there are other validated anthropometric measures available for estimating adiposity^12,13^, BMI use remains routine due to its convenience and ease in measurement that even self-reported weight and height can be used to calculate it for weight classification purposes^14^. The currently used BMI formula itself was developed more than 150 years ago by Belgian mathematician Adolphe Quetelet during a period when body fat estimation necessitated using more convenient methods that did not involve sophisticated calculations. It was referred to then as the Quetelet index until American physiologist Ancel Keys named it ‘body mass index’ in 1972. However, L. N. Trefethen, an applied mathematician and professor of numerical analysis at the University of Oxford, critiques this formula, stating that it divides the weight by too much with shorter people, and by too little with tall people^15^, consequently underestimating adiposity in the former and overestimating it in the latter. In light of this, he proposed a modified BMI formula (Eq. 1) he believes would approximate actual body sizes and shapes better^16^:

1 $$BMI=\frac{1.3(weight\,in\,kg)}{{(height\,in\,m)}^{2.5}}$$

The multiplicative factor of 1.3 is intended to make the BMI value unchanged for an adult with height of 1.69 m, selected as the average adult height in the design of the formula (the square root of 1.69 is 1.3). As one deviates from this average height, the difference between the BMIs computed using the traditional and proposed formulae correspondingly becomes more evident. In this study, we aimed to assess the performance of the BMI calculated using the modified formula (BMI_T_, the subscript ‘T’ referring to Trefethen) against that calculated using the current or traditional formula (BMI_K_, the subscript ‘K’ referring to Keys) in predicting %BF and in screening for overweight/obesity among a sample of Filipino young adults. As a secondary objective, we aimed to assess and compare the measures of diagnostic accuracy of both BMI measures in identifying the overweight-obese state.

## Results

Table 1 summarises the demographic and anthropometric data of the study participants. Of the 783 medical students in the study population that were invited, only 190 (74 males and 116 females) participated in the study. The median age of the sample was 22 years (range: 19–30 years). Only 3 students in the sample (1.6%, all males) were smokers at the time of data collection. Regarding alcohol intake, 29 (15.3%) admitted having had at least 1 drink per week (19 males vs 10 females; *p* = 0.0014). In our sample, males had significantly higher values for height, weight, WC, BMI_K_ and BMI_T_ (all *p* < 0.001), while females had significantly higher %BF (*p* < 0.001). Among males, 28 (37.8%) had %BF ≥ 25%, while among females, 21 (18.1%) had %BF ≥ 35%.

**Table 1:** Demographic and anthropometric characteristics of participants.

Characteristic	Males (n = 74)	Females (n = 116)	*p*-value
Age, median (range)	22 (19–30) years	22 (19–27) years	0.614
Height, mean (SD)	168.9 (5.0) cm	156.1 (5.7) cm	< 0.001
Weight, mean (SD)	76.1 (14.8) kg	53.2 (11.5) kg	< 0.001
WC, median (range)	88.5 (66.5–125.0) cm	73 (59.5–102) cm	< 0.001
%BF, mean (SD)	23.2 (5.5) %	29.6 (5.2) %	< 0.001
Smoking history, median (range)	0 (0–20) pack-years	0	0.029
Alcohol intake, median (range)	1 (0–10) drinks/week	0 (0–3) drinks/week	0.002
**BMI**_**K**_**, mean (SD)**	26.6 (5.0)	23.0 (4.3)	< 0.001
< 18.5 (n, %)	1 (1.3%)	13 (11.2%)	
18.5–22.9 (n, %)	19 (25.7%)	54 (46.6%)	
23.0–24.9 (n, %)	11 (14.9%)	19 (16.4%)	
≥ 25.0 (n, %)	43 (58.1%)	30 (25.9%)	
**BMI**_**T**_**, mean (SD)**	26.7 (5.0)	23.9 (4.5)	< 0.001
< 18.5 (n, %)	1 (1.3%)	4 (3.4%)	
18.5–22.9 (n, %)	20 (27.0%)	56 (48.3%)	
23.0–24.9 (n, %)	12 (16.2%)	20 (17.2%)	
≥ 25.0 (n, %)	41 (55.4%)	36 (31.0%)	

*%BF* body fat percentage, *BMI*_*T*_ BMI calculated using the proposed modified (Trefethen) formula, *BMI*_*K*_ BMI calculated using the Quetelet index popularized by keys, *WC* waist circumference.

Table 2 summarises the analysis of the differences between the median BMI_K_ and median BMI_T_ at the bottom and top 10% of the height distribution. In general, BMI_K_ values were lower relative to BMI_T_ in the bottom 10% while the reverse was observed in the top 10%. The BMI differences, however, did not reach statistical significance in our sample. Of note, the top 10% of the female height distribution (range: 163.2–170 cm) lies about the average adult height of 169 cm set for the modified BMI formula; among the females in the sample, only one had height > 169 cm. The BMI_K_–BMI_T_ difference was smallest in this particular female height range.

**Table 2:** Comparison of the body mass index (BMI) values at the bottom and top 10% of the height distribution.

Sex	Height distribution	Median BMI_K_	Median BMI_T_	Difference (BMI_K_ – BMI_T_)	*p*-value
Males	Bottom 10% (range: 155.5–162 cm)	23.4	24.3	–0.9	0.529
	Top 10% (range: 176–178.5 cm)	27.0	26.3	0.7	0.674
Females	Bottom 10% (range: 142–149 cm)	22.7	24.6	–1.9	0.165
	Top 10% (range: 163.2–170 cm)	21.9	21.8	0.1	0.707

*BMI*_*T*_ BMI calculated using the proposed modified (Trefethen) formula, *BMI*_*K*_ BMI calculated using the Quetelet index popularized by keys.

Table 3 shows the correlation matrix that includes both BMI types and %BF, stratified according to sex. High correlation exists between BMI_K_ and BMI_T_, as well as between %BF and BMI_K_, and between %BF and BMI_T_. The correlations, however, tended to be higher among females, albeit not significantly, basing on the overlapping 95% CIs.

**Table 3:** Correlation matrix of anthropometric measures, stratified by sex (95% confidence intervals in parentheses).

	Males			Females		
	BMI_K_	BMI_T_	%BF	BMI_K_	BMI_T_	%BF
BMI_K_	1.000			1.000		
BMI_T_	0.997 (0.995, 0.998)	1.000		0.995 (0.993, 0.997)	1.000	
%BF	0.785 (0.677, 0.860)	0.782 (0.672, 0.858)	1.000	0.833 (0.769, 0.883)	0.815 (0.745, 0.870)	1.000

*%BF* body fat percentage, *BMI*_*T*_ BMI calculated using the proposed modified (Trefethen) formula, *BMI*_*K*_ BMI calculated using the Quetelet index popularized by keys.

The crosstabulations in Table 4 show the degrees of agreement between weight classifications based on BMI_K_ and BMI_T_. The proportion of agreement was significantly lower among females (81.0%; 95% CI 73.9%, 88.2%) compared to males (96.0%; 95% CI 91.5%, 100.0%), despite the agreement statistic being substantial (κ = 0.7139; 95% CI 0.5969, 0.8309). On the other hand, there was almost perfect agreement between the two weight classifications among males (κ = 0.9306; 95% CI 0.7658, 1.0000).

**Table 4:** Contingency tables featuring degrees of agreement between weight classifications based on the BMIs computed using the Quetelet index popularized by keys (BMI_K_) and modified formula proposed by Trefethen (BMI_T_), stratified by sex.

BMI_K_	BMI_T_				
	Underweight	Normal	Overweight	Obese	Total
**Males**					
Underweight	1	0	0	0	1
Normal	0	19	0	0	19
Overweight	0	1	10	0	11
Obese	0	0	2	41	43
Total	1	20	12	41	74
Agreement: 96.0% (95% CI 91.5%, 100.0%); expected agreement: 41.6%; *p* < 0.001; Cohen’s κ: 0.9306 (95% CI 0.7658, 1.0000)					
**Females**					
Underweight	4	9	0	0	13
Normal	0	47	7	0	54
Overweight	0	0	13	6	19
Obese	0	0	0	30	30
Total	4	56	20	36	116
Agreement: 81.0% (95% CI 73.9%, 88.2%); expected agreement: 33.7%; *p* < 0.001; Cohen’s κ: 0.7139 (95% CI 0.5969, 0.8309)					

For both sexes, linear and quadratic relationships between %BF and both BMI values (BMI_K_ and BMI_T_) were analysed using robust polynomial regression, as discussed earlier. The resultant Wald *F* statistics when setting the squared BMI term to zero were statistically significant for both BMI_K_ (males: *F*_1,65.0_ = 4.81, *p* = 0.032; females: *F*_1,107.7_ = 16.37, *p* < 0.001) and BMI_T_ (males: *F*_1,65.0_ = 4.23, *p* = 0.044 ; females: *F*_1,107.7_ = 14.81, *p* < 0.001), indicating that the squared BMI term is a necessary predictor of %BF.

**Figure 1:**
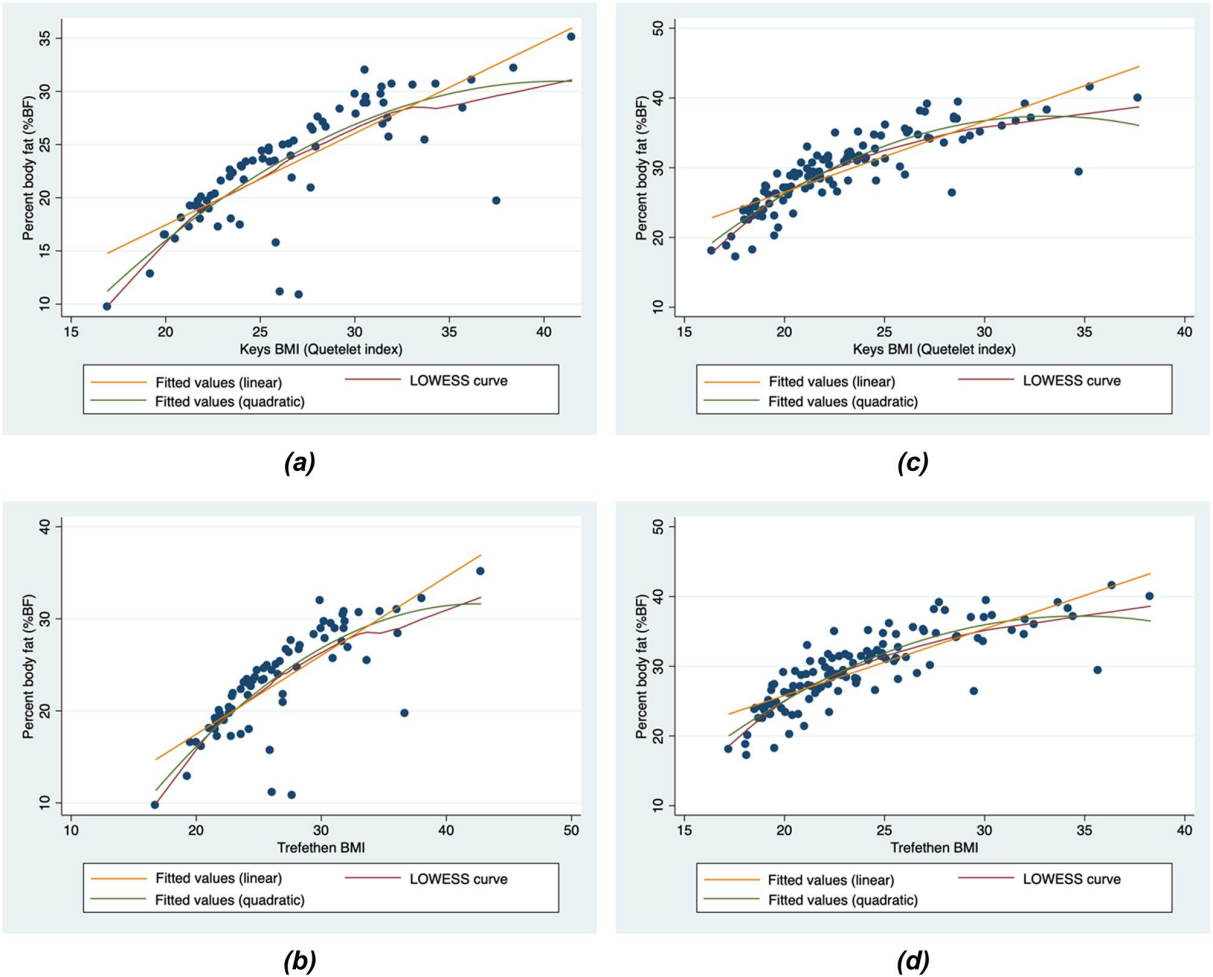
Scatterplots of BMI and %BF values per BMI measure type among males **(a,b)** and females **(c,d)** with LOWESS (locally weighted scatterplot smoothing) curves and fitted straight lines and fitted quadratic curves. *BMI* body mass index, *%BF* body fat percentage.

Regression diagnostics and model adequacy assessment were performed on all constructed sex-specific regression models. The *p*-value for the *F* statistic of each constructed model is < 0.0001, indicating that each model has significant predictive capability. All adjusted *R*^2^ values are greater than 0.50, indicating a large percentage in the variation in %BF explained by the predictors. The adjusted *R*^2^ values of all models among females were consistently higher than those among males. Additionally, both BMI values and WC were significant predictors of %BF (Table 5) among females, as opposed to the models for males wherein only BMI values significantly predicted %BF. The BMI quadratic models had higher adjusted *R*^2^ values relative to the BMI linear models for both males and females. In terms of model quality, on the other hand, the quadratic models had the lowest AIC and BIC values relative to the linear models for both sexes, indicating the former to have better fit for the data. Similarly, the BMI_K_ quadratic models had the highest adjusted *R*^2^ values and the lowest AIC and BIC values relative to the BMI_T_ quadratic models, also for both males and females, indicating that the BMI_K_ quadratic models have better fit for the data compared to the BMI_T_ quadratic models. The adjusted *R*^2^, AIC and BIC values for the different models are summarised in Table 6. Independence of the error terms was demonstrated on residual analysis of each of the constructed models. However, the LOWESS curves (fitted to assess linearity between BMI and %BF in each constructed model) visually depart from the straight line fitted through the %BF-versus-BMI data points, better approximating the fitted quadratic curves instead (see Fig. 1). Furthermore, the BMI_K_ and BMI_T_ linear models for both sexes failed both model specification and omitted variables tests, indicating that the correct forms of the predictor or certain omitted variables were most likely not included in said models. The opposite is true for the corresponding quadratic models, indicating that the squared BMI term appears to be the more appropriate form of the main predictor variable (BMI) with explanatory power to add to said models. Overall, assessment of model adequacy and quality favors the quadratic models over the linear models.

**Table 5:** Summary of the robust polynomial regression analysis of the BMI_K_ and BMI_T_ quadratic models.

Variable	Observed coefficient, B	95% confidence interval	Robust standard error	Z	*p*-value
**BMI**_**K**_** quadratic model (males)**					
(Constant)	–25.861	–45.876, –5.848	10.007	–2.58	0.012
BMI_K_	2.918	0.941, 4.895	0.990	2.95	0.004
BMI_K_^2^	–0.035	–0.067, –0.003	0.016	–2.19	0.032
Age	0.010	–0.427, 0.445	0.217	0.04	0.968
Smoking	–0.046	–0.156, 0.065	0.055	–0.82	0.414
Alcohol intake	0.144	–0.106, 0.395	0.125	1.15	0.254
WC	–0.038	–0.255, 0.179	0.109	–0.35	0.729
**BMI**_**T**_** quadratic model (males)**					
(Constant)	–23.726	–43.121, –4.331	9.696	–2.45	0.017
BMI_T_	2.529	0.695, 4.362	0.918	2.75	0.008
BMI_T_^2^	–0.030	–0.058, –0.001	0.014	–2.06	0.044
Age	0.033	–0.410, 0.476	0.220	0.15	0.882
Smoking	–0.057	–0.172, 0.057	0.057	–1.00	0.322
Alcohol intake	0.181	–0.079, 0.441	0.130	1.39	0.169
WC	0.004	–0.212, 0.220	0.108	0.04	0.971
**BMI**_**K**_** quadratic model (females)**					
(Constant)	–38.783	–54.944, –22.621	8.152	–4.76	< 0.001
BMI_K_	3.338	1.947, 4.729	0.702	4.76	< 0.001
BMI_K_^2^	–0.055	–0.082, –0.028	0.014	–4.05	< 0.001
Age	0.194	–0.044, 0.432	0.120	1.62	0.108
Smoking	0	Omitted (no observations)			
Alcohol intake	–0.071	–0.685, 0.542	0.309	–0.23	0.818
WC	0.233	0.104, 0.362	0.065	3.59	< 0.001
**BMI**_**T**_** quadratic model (females)**					
(Constant)	–38.028	–54.535, –21.521	8.326	–4.57	< 0.001
BMI_T_	2.864	1.581, 4.147	0.647	4.43	< 0.001
BMI_T_^2^	–0.048	–0.072, –0.023	0.012	–3.85	< 0.001
Age	0.189	–0.055, 0.433	0.123	1.54	0.128
Smoking	0	Omitted (no observations)			
Alcohol intake	0.049	–0.570, 0.668	0.312	0.16	0.876
WC	0.307	0.194, 0.419	0.057	5.41	< 0.001

*%BF* body fat percentage, *BMI*_*T*_ BMI calculated using the proposed modified (Trefethen) formula, *BMI*_*K*_ BMI calculated using the Quetelet index popularized by keys, *WC* waist circumference.

**Table 6:** Summary of adjusted *R*^2^ values, Akaike information criterion (AIC) and Bayesian information criterion (BIC) of various sex-specific models regressing %BF on BMI values.

Model	Adjusted *R*^2^		AIC		BIC	
	Males	Females	Males	Females	Males	Females
BMI_K_ linear model	0.5938	0.7478	399.68	557.96	413.51	571.73
BMI_K_ quadratic model	0.6300	0.8094	393.66	526.42	409.79	542.94
BMI_T_ linear model	0.5930	0.7442	399.83	559.60	413.65	573.37
BMI_T_ quadratic model	0.6205	0.7935	395.55	535.71	411.68	552.24

*BMI*_*T*_ BMI calculated using the proposed modified (Trefethen) formula, *BMI*_*K*_ BMI calculated using the Quetelet index popularized by keys.

Table 7 lists the sensitivity, specificity, predictive values, and likelihood ratios of BMI_K_ and BMI_T_ in diagnosing overweight–obesity by %BF (defined as ≥ 25% in males and ≥ 35% in females), using BMI of 23 as cutoff between the normal and overweight–obese categories. Consistently, performance was excellent in terms of sensitivity and negative predictive value, but with significantly lower specificity and positive predictive value. Of note, positive predictive value of BMI among females, regardless of BMI type, was significantly lower than that among males.

**Table 7:** Summary of measures of accuracy of BMI_K_ and BMI_T_ in diagnosing overweight–obese.

Measure	BMI_K_		BMI_T_	
	Males	Females	Males	Females
Sensitivity	100% (87.7%, 100%)	95.2% (69.5%, 99.9%)	100% (87.7%, 100%)	95.2% (76.2%, 99.9%)
Specificity	44.4% (29.6%, 60.0%)	69.5% (59.2%, 78.5%)	46.7% (31.7%, 62.1%)	62.1% (51.6%, 71.9%)
Positive predictive value (PPV)	58.2% (38.6%, 66.7%)	40.8% (27.0%, 55.8%)	53.8% (39.5%, 67.8%)	35.7% (23.4%, 49.6%)
Negative predictive value (NPV)	100% (83.2%, 100%)	98.5% (92.0%, 100%)	100% (83.9%, 100%)	98.3% (91.1%, 100%)
Likelihood ratio ( +)	1.77 (1.36, 2.31)	3.03 (2.2, 4.18)	1.85 (1.4, 2.43)	2.45 (1.85, 3.24)
Likelihood ratio (–)	0.039 (0.002, 0.615)	0.098 (0.021, 0.464)	0.037 (0.002, 0.586)	0.11 (0.023, 0.520)

The 95% CIs are indicated in parentheses. Overweight–obesity is defined as ≥ 25% BF in males and ≥ 35% body fat in females. The PPV and NPV were adjusted for sample prevalence of overweight-obese based on body fat percentage.

*BMI*_*T*_ BMI calculated using the proposed modified (Trefethen) formula, *BMI*_*K*_ BMI calculated using the Quetelet index popularized by keys.

**Figure 2:**
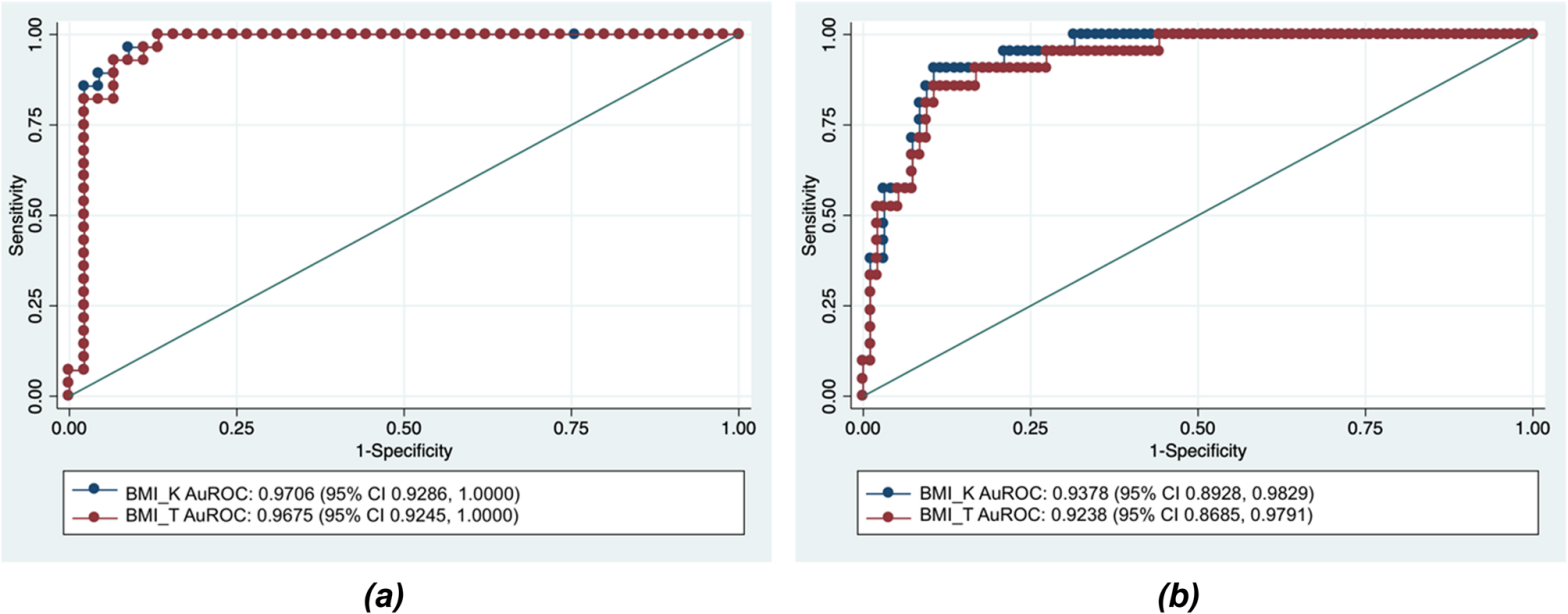
Receiver-operator characteristic (ROC) curves with corresponding areas under the curves (AuROCs) for BMI_K_ and BMI_T_ among males **(a)** and females **(b)**. The 95% CIs are indicated in parentheses. *BMI*_*T*_ proposed modified (Trefethen) BMI, *BMI*_*K*_ traditional Quetelet BMI.

The optimal BMI_K_ cutoff was identified at 26.7 for males (Sn = 96%, Sp = 91%) and 25.1 for females (Sn = 90%, Sp = 89%), while for BMI_T_, the optimal cutoff was 26.3 for males (Sn = 100%, Sp = 87%) and 26.3 for females (Sn = 86%, Sp = 89%). Figure 2 shows the sex-specific ROC curves for both BMI_K_ and BMI_T_. All ROC curves had optimised AuROCs > 0.90, indicating high diagnostic accuracy, and were significantly higher than the corresponding BMI_K_ and BMI_T_ AuROCs where BMI cutoff of 23.0 was used (0.7220 and 0.7330 respectively among males, 0.8240 and 0.7870 among females). The optimised AuROC for BMI_K_ was slightly higher than that for BMI_T_, but the difference was not statistically significant.

Because the target sample size was not achieved, post-hoc power analysis was performed. In line with the study’s primary objective, Cohen’s *f*^2^ effect sizes were determined using the increase in adjusted *R*^2^ (Δ*R*^2^_adj_) computed from the actual study sample after including BMI as predictor of %BF (i.e., the additional percentage of the variation in %BF explained by BMI). The Δ*R*^2^_adj_ attributed to the addition of both BMI and BMI^2^ terms to the reduced model (i.e., the model containing only age, smoking status, alcohol intake and WC as predictors) was used because the BMI_K_ and BMI_T_ quadratic models demonstrated better fit to the data, as evidenced by their lower AIC and BIC values, compared to the linear models. According to Cohen’s guidelines^17^, *f*^2^ ≥ 0.02, *f*^2^ ≥ 0.15 and *f*^2^ ≥ 0.35 correspond to small, medium and large effect sizes, respectively. As stated under ‘Methods,’ a minimal detectable effect size of 0.025 was assumed. Overall, the actual effect sizes in this study are larger than the expected minimal detectable effect size. The adjusted *R*^2^ values for the sex-specific reduced models are 0.4965 for males and 0.7330 for females. Among males, the actual Δ*R*^2^_adj_ values (and effect sizes) are 0.1335 (*f*^2^ = 0.154) and 0.1240 (*f*^2^ = 0.142) for BMI_K_ and BMI_T_, respectively. Given these Δ*R*^2^_adj_ values, the obtained sample size of 74 for males, and α = 0.05, corresponding powers of 99.70% and 99.39% were achieved, respectively. On the other hand, the actual Δ*R*^2^_adj_ values (and effect sizes) for the BMI_K_ and BMI_T_ quadratic models among females are 0.0764 (*f*^2^ = 0.083) and 0.0605 (*f*^2^ = 0.064), respectively. Given these Δ*R*^2^_adj_ values, the obtained sample size of 116 for females, and α = 0.05, the respective achieved powers of 99.99% and 99.97% were computed. Basing on these power calculations, the number of study participants is deemed sufficient for each sex-specific (quadratic) predictive model.

On the other hand, the actual sample size underpowered the study with respect to a part of the secondary objective. More specifically, despite the ROC curves individually having high statistical power (99.99% for each of the four ROC curves generated) in terms of discriminating between the normal and overweight-obese states (most likely attributed to their high optimised AuROCs), the calculated achieved powers for testing the evidence against the null hypothesis that the AuROCs for BMI_K_ and BMI_T_ are the same are 24.45% for males and 10.96% for females.

## Discussion

The Philippines is a middle-income level country that has moved from an agriculture-based economy to a more manufacturing-based one in the recent years, having an average GDP growth that increased from 4.5% between 2000 and 2009 to 6.3% from 2010 through 2017^18^. As of 2018, its economic growth was pegged at 6.2%, and is forecasted at 6.4% in 2019 and 2020^19^. The country likewise has a large proportion of young people, placing the labor force participation rate at 60.2% as of January 2019^20^. Concurrent with the Philippines’ growing industrialisation, however, the Filipino diet has progressively moved towards that consisting of processed meats and foods containing high-fructose corn syrup, on top of consuming refined white rice as staple. With majority of the workplace becoming increasingly sedentary, presumably due to more office-based job opportunities, many young adults are at risk for obesity, a condition which most health professionals screen for and diagnose by computing the BMI.

While being a widely used and inexpensive anthropometric measure, BMI has its share of criticisms and drawbacks, particularly important of which is its inability to differentiate between fat and lean body mass, arguably making it an indicator of heaviness rather than adiposity. This is exemplified by physically active occupational groups (policemen, firefighters, athletes), all having considerably greater muscle mass and consequently higher BMIs despite very low %BF^21^. While squaring the formula’s height term in the denominator supposedly adjusts body composition to height^22^, analyses of samples from several diverse populations failed to demonstrate independence of BMI (and by extension, %BF) from height^23^. Moreover, BMI underestimates adiposity in individuals with smaller frames while overestimating it in tall people. López-Alvarenga et al.^24^ showed that short-statured individuals (women ≤ 1.50 m, men ≤ 1.60 m) had significantly higher %BF compared to their taller sex-, age- and BMI-matched counterparts, with wider differences at BMI ≥ 25. In light of increasing obesity rates in Southeast Asia, this becomes pertinent to the Filipino population, whose average height approximates the short-stature range^25^. Conversely, tall people tend to have narrower builds, with a larger proportion of their body components being skeletal muscle and bone, and their legs carrying a larger proportion of their weight. This results in having higher lean-to-fat ratios compared with short-statured individuals^26^.

Believing that the conventional BMI leads short-statured individuals to think they are thinner than they are, and tall people to think the opposite, L. N. Trefethen proposed a modification to the BMI formula by raising height to a power of 2.5 instead of squaring it. Although no epidemiological evidence supports using such an exponent on height^27^, which Trefethen himself disclosed, he explained that using an exponent of 3, as would be the case if weight scaled up the same manner as height^23,26^, would not fit the data well if people’s weights were plotted against their heights. He added that a better fit would be obtained if height was raised to 2.5 instead^28^. The multiplicative factor of 1.3 is based on the square root of 1.69 m, which was set as the average height for adults in the design of this new formula^16^. At this height, the BMI_K_ and BMI_T_ are equal. If weight was kept constant and BMI_K_ and BMI_T_ were to be plotted against height, the respective downsloping curves will intersect at 1.69 m, with the BMI_T_ graph steeper than the BMI_K_ graph. Naturally, this results in a very high correlation between BMI_K_ and BMI_T_, as was observed in our study. Thus, for individuals < 1.69 m, BMI_T_ is larger than BMI_K_, with the opposite observed for heights > 1.69 m.

We observed high correlations between BMI and %BF in our study, with the correlations being non-significantly higher among females. This noted sex difference has been demonstrated in prior observational studies^29–31^ and can be explained by the greater fat content among women compared to men for any given BMI^32^, as well as greater lean mass and bone density among males. Males with higher BMIs would tend to have lower %BF compared to females with the same BMI range since skeletal muscle is relatively denser than adipose tissue, again stressing the inability of BMI to distinguish between lean and fat mass. Despite similar correlations with %BF, however, there was greater discordance between BMI_K_ and BMI_T_ among females (average BMI_T_ is almost 1 point higher than average BMI_K_, compared to only a 0.1 difference observed among males), which lead to lower agreement between the two BMI measures in this group, reflected by a step-up in weight classification among 69.2%, 13.0%, and 31.6% of females classified initially as underweight, normal, and overweight, respectively, using BMI_K_. Consistent with the consequence of the modified formula’s design, the sole female in our sample with height > 1.69 m had BMI_K_ larger than BMI_T_, while the converse was true for the rest. Of note as well, only males in our sample had step-downs in weight classification (i.e., having lower mean BMI_T_ compared to BMI_K_). This occurrence has been observed in two prior studies involving surgical patients that also evaluated BMI_T_ against BMI_K_, albeit using postoperative complications^33,34^ and long-term survival^34^ as outcomes. Such observation is attributable to males having generally greater height than females on average, upholding Trefethen’s conjecture that adiposity might be overestimated in tall individuals. It is then tenable to consider that such shifts in weight classifications may have important implications for long-term mortality among individuals with pre-existing comorbidities (e.g., hypertension, diabetes mellitus, COPD, cardiovascular disease, malignancy) or for development of these same comorbidities over time among younger individuals who are disease-free at baseline. The questions now would be if shifting towards the optimal weight classification based on BMI_T_ would translate to more favorable long-term outcomes, given the premise that BMI_T_ may be better in terms of approximating actual body sizes and shapes. Data available to date^34,35^ do not support the presence of any long-term outcome advantage with one BMI measure over the other in populations with concomitant chronic medical conditions. Wang et al.^35^ reported no significant differences in long-term graft survivals between BMI_T_ and BMI_K_ among a cohort of living-related kidney transplant recipients (mean follow-up time: 72.9 months), however, the study is limited by a small sample size. Similarly, in a study by Tjeertes et al.^34^ involving a large cohort of surgical patients, comparable results between BMI_T_ and BMI_K_ in predicting long-term survival were demonstrated after adjusting for age, sex, surgical risk, type of anesthesia, ASA classification and medical comorbidities (median follow-up time: 6.3 years). However, in the same vein, whether BMI_T_ would be better than BMI_K_ in predicting the risk of incident chronic medical illnesses among young disease-free individuals over a follow-up period significantly longer than 10 years, or whether BMI_T_ would model long-term survival better in this particular population, has not yet been investigated to the best of our knowledge, and indeed would be something worth looking into. Since the BMI–%BF relationship changes with age, the effect of each BMI measure on such long-term outcomes can be modeled comparatively while taking age as time-dependent covariate into consideration.

In the construction of our sex-specific regression models, the following factors associated with %BF were included as covariates: age^36,37^, smoking^38,39^, alcohol intake^40,41^, and WC^42^. Available data regarding the shape of the BMI–%BF relationship (that is, whether it is linear^7,43^ or quadratic^44,45^) are conflicting, and perhaps can be attributed to observable differences in regional mass or body composition proportions between races or ethnic groups^46,47^. In our study, the quadratic models fitted the data best for both sexes, basing on model adequacy assessment and information criteria. This is consistent with studies demonstrating the BMI–%BF relationship to be quadratic among Asians^45,48,49^, and has been attributed to their generally shorter stature and higher %BF despite normal BMIs^50–52^. This quadratic association, however, appears to be weaker at lower BMI ranges^45,49^. In their study on a group of Sri Lankan adults, Ranasinghe et al.^48^ demonstrated a statistically significant adjusted *R*^2^ increase after addition of the BMI^2^ term to each of their sex-specific regression models. This finding is similar to the significant Wald tests we obtained from assessing the goodness of fit of the linear and quadratic models for each BMI type.

Differences among ethnic groups have likewise been observed in terms of how BMI corresponds with %BF. In a meta-analysis of 11,924 study participants^46^, various ethnic groups demonstrated lower average BMI for the same %BF relative to their age- and sex-matched Caucasian counterparts. As the risk for developing and dying from weight-related chronic medical conditions generally increases with increasing adiposity (as estimated by BMI), these results imply that even at relatively lower BMIs, such risk may be significantly higher for the involved ethnic populations. Within a given range of BMI values, Asians were found to be at greater risk for hypertension, dysglycemia/diabetes mellitus and dyslipidemia compared to Caucasians^53,54^. Using this same comparison, Asians were likewise found to have higher all-cause mortality risk starting at BMI ≥ 25.0, five points lower than the Caucasian BMI cutoff for overweight^55^. While the WHO has addressed this excess risk in 2004 by recommending lower overall BMI cutoffs for weight categories in Asian populations^56,57^, no specific cutoff points for each population were redefined, citing several emerging candidate cutoff values corresponding to population-specific health risks observed from the available data. For that matter, some Asian countries went on to define and adapt their own BMI ranges for overweight and obese that they have already used as triggers for health policy action^58^. Given these circumstances, it may seem like an attractive proposition to construct country-specific BMI formulas, but their design can be complicated. For a start, it is unclear how the BMI–%BF correspondence can be factored into the formula. However, going back to the design of Trefethen’s formula, we recall that 1.69 m is selected as the average adult height, and by using its square root (1.3) as the multiplicative factor, both BMI_T_ and BMI_K_ are unchanged for a 1.69-m tall individual. This formula can be similarly fashioned such that the country-specific averages for adult heights are used instead of 1.69 m, and empirical data collected to test hypotheses about the behaviour of the BMI–%BF relationship. Centering the BMI formula to a country’s average height does not have any epidemiological basis like Trefethen’s formula, but these are potential exploratory attempts at describing and explaining the complex behaviour of the BMI–%BF relationship. To our knowledge, these suggested modifications to the BMI formula have not yet been investigated. Pilot studies are still necessitated to assess which of these formulae can optimally predict %BF, followed by compulsory validation in larger subsequent studies.

Also, in our study, WC was found to be a significant predictor of %BF, but only among females. It has long been recognised that certain Asian ethnic groups have high prevalence of abdominal adiposity^56^, putting them at risk for metabolic syndrome. Apart from women possessing greater fat content than men for any given BMI, as discussed earlier, Asian women in particular tend to have greater abdominal and visceral adiposity^59,60^. In a study that looked into ethnic differences in abdominal adiposity, Filipino women were found to have the highest visceral adipose tissue content compared to their BMI- and WC-matched Caucasian and African American counterparts^61^. In the Philippine setting, indicators of socioeconomic status were found to have a positive relationship with central obesity among young adults, particularly among women, living in the lower-income range^62^. This clearly contradicts what is commonly observed about men tending to accumulate more abdominal fat, and women tending to accumulate fat in the thigh and gluteal areas. While the definite reason for this remains unknown, researchers posit a lower capacity of Asians to store fat subcutaneously to be responsible^59^. However, this may as well be a reflection of increasing industrialisation and the change in dietary habits that accompanies it.

Using %BF cutoff values of 25% for males and 35% for females, and a BMI cutoff of 23.0 for both sexes to distinguish between normal and overweight-obese categories, both BMI_K_ and BMI_T_ had comparable measures of diagnostic accuracy in terms of %BF. While the two BMI measures performed well in terms of sensitivity and negative predictive value based on our data, both had comparably poor specificities, particularly among males. This finding has been observed in similar prior studies^52,63,64^, and highlights the inherent inability of BMI to distinguish between lean mass and adipose tissue even despite restricting the sample to nonathletic individuals. Of equal interest is the noticeably poor positive predictive value, particularly among females, indicating that > 50% of them with BMI ≥ 23.0 did not satisfy the criteria for being overweight-obese by %BF. When we optimised the BMI_K_ and BMI_T_ cutoffs, all generated ROC curves demonstrated high accuracy at AuROCs > 0.90. We warn, however, that even though high statistical power was individually achieved by each ROC curve in terms of discriminating between the normal and overweight-obese state, our study is underpowered for one aspect of our secondary objective, which is to determine if a statistically significant difference exists between the BMI_K_ and BMI_T_ AuROCs. For now, we caution against generalising that one BMI measure is not any better than the other in terms of diagnostic accuracy, as the true state of affairs may state otherwise. Notwithstanding this, we deemed it still important to report such measures, as they may be useful in providing a rough estimate of the diagnostic performance of each BMI measure, naturally with the caveat that these results are replicated in subsequent studies with sample sizes that are sufficient for such purpose. Additionally, it is interesting to point out that a number of studies involving nonathletic adults that used ROC analysis to diagnose the overweight-obese state^30,65–67^ demonstrated similar optimal Keys BMI (Quetelet index) values within the range of 24.0 to 28.0, with AuROCs that are at least in the acceptable range. Fortuitously, it is approximately in this BMI range in the general population where its inherent incapacity to distinguish lean mass from adipose composition is probably higher^63,68^. Comparing these results with our data, however, becomes complicated due to the different methods used in measuring %BF and the absence of standard %BF values for qualifying excess adiposity. So far, all seem to suggest the need to reevaluate the utility of current BMI cutoffs used in obesity screening and health policy making^69^, as well as the need to establish a diagnostic %BF cutoff in light of results from long-term prospective studies involving Asian populations that investigate health outcomes as a function of BMI or %BF.

In this study, not only were we able to evaluate the ability of Trefethen’s modified BMI formula against the traditional Quetelet index in predicting %BF, we also assessed their overall performances in discriminating between normal and overweight-obese weight classifications by means of AuROCs using sex-specific %BF cutoff values. To the best of our knowledge, this is the first study to do so with these particular objectives. Perhaps one strength of this study is that recruitment was limited to young adults, which minimised the effect of any age-related variation in body composition. Similarly, we restricted our sample to nonathletic individuals, thus avoiding inclusion of participants with large BMIs yet small %BF that could excessively inflate false-positive rates. We likewise restricted study participation to volunteers who are of Filipino descent, which minimised the probability of recruiting a sample that is heterogeneous in terms of body proportions and composition, thereby avoiding any potential wide within-group variations in BMI and %BF measurements. We also made use of robust polynomial regression in our analysis; compared to conventional multiple regression analysis, it provides better regression coefficients even in the presence of violations to the normality and homoscedasticity assumptions. Additionally, we were able to show how the sex-specific quadratic regression models explained the BMI–%BF relationship better relative to their corresponding linear regression models by means of model quality and adequacy assessment. Lastly, we demonstrated how the weight classification of some participants changed following computation of BMI_T_. While examining the association between BMI_T_ and long-term health outcomes is beyond the scope of this study, further prospective studies can be undertaken to determine if this change in weight classification has long-term health implications, as discussed earlier.

Our study also has its share of limitations. Because participation was on a voluntary basis, self-selection bias was inevitably introduced, possibly contributing to an overrepresentation of the overweight and obese weight categories in the sample. In addition to the non-probability nature of voluntary sampling, this carries potential implications when determining measures of diagnostic accuracy, as some are quite sensitive to the prevalence of the condition of interest (the overweight-obese state). In our sample, the proportions of overweight and obese participants were indeed larger than the prevalence indicated in the national statistics^4^. To address this, adjustments for overweight-obese prevalence in the study sample were made in computing for the predictive values. The small sample size we obtained also contributed to this study’s limitations. Even though our study was adequately powered for the primary objective, our obtained sample size underpowered this study with respect to the part of the secondary objective involving testing the evidence against the null hypothesis that the AuROCs for BMI_K_ and BMI_T_ are the same. Despite this, we still obtained results comparable with those obtained in similar prior studies, as discussed previously. We, however, still caution against generalising that one BMI measure is not any better than the other in terms of diagnostic accuracy until these results have been replicated in subsequent studies that are better powered for this purpose. While we discussed how the restrictiveness of our study criteria helped minimise possible confounding and/or effect modification by age, our study is also limited in that our results are not generalisable to older individuals. However, staying faithful to our study’s statement of the problem, our primary focus is on young Filipino adults, with whom the increasing obesity prevalence rates are a public health concern. Also, because to our study criteria, our results cannot be generalised to physically active occupational groups and non-Filipinos. Similarly, because all study participants were free of conditions such as hypertension, diabetes, dyslipidemia, COPD and cancer at the time of study inclusion, the effect of such comorbid chronic illnesses on the BMI–%BF relationship cannot be accounted for in our results. As discussed earlier, whether BMI_T_ can better predict the long-term risk of incident chronic medical illness among a cohort of young disease-free adults can be a potential topic for research. Next, whereas a quadratic relationship between BMI and %BF has been demonstrated in our study, with this relationship similarly observed among Asian populations, it remains difficult to construct individualised country-specific BMI formulae that aim to extrapolate %BF predictions for each country without first collecting empirical data for purpose of validating such formulae. Finally, our study made use of bioelectric impedance analysis (BIA) in quantifying %BF. While dual energy x-ray absorptiometry (DXA) has become more of a “gold standard” in assessing body composition over the past decade, its cost still limits its routine use. As a cheaper alternative, BIA indirectly estimates body adiposity by estimating fat-free body mass (by estimating total body water) through the use of electrical impedance and subtracting it from total body weight. It is found to be reliable for use in epidemiological studies^70,71^, provided the necessary preparations are made prior to use (i.e., observing proper body position and avoidance of physical exercise and food or fluid intake beforehand).

## Conclusion and recommendations

Based on our study on a sample of young Filipino adults, both BMI_K_ and BMI_T_ significantly predict %BF, as well as adequately discriminate between %BF-defined normal and overweight-obese states using optimal BMI cutoff values. Both BMIs, however, performed poorly in terms of specificity, indicating that even with the modifications afforded to the Quetelet index, BMI_T_ is unable to differentiate between fat and lean mass. Given how shifting from BMI_K_ to BMI_T_ resulted in changes in weight classification among participants < 1.69 m tall, we recommend that further prospective studies be undertaken to determine if such changes have long-term health implications.

## Methods

### Study design

This is a cross-sectional observational study that was carried out during the second semester of academic year 2018–2019 at the De La Salle Medical and Health Sciences Institute (DLSMHSI), City of Dasmariñas, Cavite Province, Philippines. Ethics approval of the study was granted by the Institutional Ethics Review Committee of the College of Medicine of DLSMHSI, in accordance with the institution’s ethical guidelines for observational studies.

### Study population

The study population comprised of medical students enrolled at the DLSMHSI. They are composed of young Filipino adults originating from various areas in the Philippines (though predominantly from the Central Luzon and CALABARZON regions) who came to enroll in the institute’s medicine program. Given the presumed high level of stress associated with medical school and the relatively sedentary lifestyle of the average medical student, it was anticipated that the prevalence of overweight and obesity in this population will be at least comparable with that indicated in the national statistics^4^.

All first- to third-year students duly enrolled in the medicine program of DLSMHSI were considered for possible participation in the study. Fourth-year students were not included because of their limited accessibility due to their full-time hospital duties. For the academic year 2018–2019, there were 262 first-years, 280 s-years, and 241 third-years, bringing the study population size to 783.

### Sample size computation

During the design of the study, a priori sample size determination for the primary objective was performed using multiple linear regression as basis, taking the constraint of the study population size (N = 783) into consideration. Due to the lack of prior studies reporting specific increments in adjusted *R*^2^ (Δ*R*^2^_adj_) following addition of BMI as predictor of body fat percentage, different candidate sample size estimates per sex-specific regression model were considered using assumed small Cohen’s *f*^2^ effect sizes ranging from 0.02 to 0.14 (corresponding to *R*^2^ deviations from zero ranging from 0.0197 to 0.1229). Fixing the number of predictors to 6 (namely age, smoking status, alcohol intake, waist circumference, BMI and BMI^2^), and assuming an α of 0.05 and a power of 80%, candidate sample size requirements ranging from 72 to 480 were calculated. Considering the constraint placed by the study population size, with a roughly 1:1 male-to-female ratio, an expected effect size of *f*^2^ = 0.025 (the minimal detectable effect) was selected to maximise the sample size requirement to 376 per sex-specific regression model.

### Participant recruitment

The invitation to participate was extended to all members of the study population. In order to reach the students, the research team made classroom visits during which a brief overview and explanation of the study objectives and procedures were presented. Participation in the study was on a voluntary basis, and a participant was deemed eligible if they were between 18 and 35 years of age at the time of recruitment and of Filipino descent. The presence of any of the following at the time of recruitment warranted exclusion: chronic illness (diabetes mellitus; hypertension; heart failure; malignancy), acute myocardial infarction or stroke within the past 6 months, pregnancy (for females), chronic corticosteroid use, conditions affecting posture (kyphosis, scoliosis, or kyphoscoliosis), or active engagement in any body-building or exercise program. All eligible participants were informed about the details of the study procedure, as well as the study’s risks, benefits and potential impacts. After having received and understood all pertinent information related to the research, their informed consent was obtained and they were provided with written consent forms thereafter for them to sign. Those who did not give their informed consent for participation were excluded from the study accordingly. No incentives were offered for participation.

### Data collection

Data collection was performed by direct interview and direct measurement. To facilitate this, the entire group of eligible participants was broken down into batches. The batches were then instructed to proceed to the College of Medicine skills laboratory at scheduled times for anthropometric measurements, so that data collection would not interfere with their classes. Booths in the skills laboratory were utilised to ensure privacy of the participants during measurements. All participants were instructed not to eat or drink anything at least two hours prior to their scheduled measurement times to avoid obtaining spurious readings with bioelectric impedance analysis (BIA). Prior to anthropometrics measurement, participants were asked for fill out a questionnaire inquiring about their demographic data, smoking history (quantified as pack-years) and alcohol intake (quantified as average number of drinks per week). All digital equipment were calibrated prior to use and regularly throughout the entire process of data collection. All measurements were taken twice by two members of the research team.

For height measurement, a verified height rule was mounted on a hard flat wall surface with its base at floor level. To check its proper vertical placement, a carpenter’s level was used. Participants were asked to remove their shoes and any heavy outer clothing. Hairstyles were adjusted or undone, and hair accessories removed to allow for proper placement of the stadiometer head piece. Participants were then instructed to stand (with back to the height rule) as straight as possible with arms hanging loosely at their sides and feet flat on the floor. The stadiometer head piece was then placed in position, and the height recorded to the nearest 0.1 cm.

Weight and %BF were measured using a digital weighing scale and body composition monitor (Tanita BC-543 One Size). The instrument was placed on a flat hard-floor (non-carpeted) surface verified using a carpenter’s level. As with height measurement, participants were instructed to remove their shoes and any heavy outer clothing, as well as empty their pockets and remove any jewelery, watches, and other accessories they were wearing. Since BIA was used to quantify %BF, participants were asked to stand barefoot on the footplates of the weighing scale. Weight was recorded to the nearest 0.1 kg, while %BF was recorded to the nearest 0.1 percent.

Waist circumference (WC) was measured using a standard tape measure, the length of which was verified regularly using a calibrated length rod. Stretched-out tape measures were replaced accordingly. The participants were asked to remove their upper garments except for light clothing that can be lifted up to the epigastric level. The midpoint between the subcostal margin of the last palpable rib and the upper margin of the iliac crest was used as anatomic landmark on which the tape measure is firmly held and maintained in horizontal position. All measurements were recorded to the nearest 0.1 cm.

The measured heights and weights were then used to calculate BMI using both the traditional formula popularised by Keys in 1972 (BMI_K_ = weight in kg/(height in m)^2^) and the proposed modified formula by Trefethen (Eq. 1). Separate weight classifications according to Asian-Pacific cutoff points^56^ (underweight: < 18.5; normal: 18.5–22.9; overweight: 23.0–24.9; obese: ≥ 25.0) were determined for each participant using the two calculated BMI values. For %BF, values of ≥ 25% in males and ≥ 35% in females were used to define obesity^72^.

A case report form was created for each participant, where all demographic information (age, sex, smoking history, alcohol intake) and raw measurements (weight, height, %BF, WC) were recorded and verified concomitantly. The data were then encoded according to the instructions specified in the coding manual, and inputted to Microsoft^©^ Excel in a data layout format appropriate for importing to the statistical software.

### Statistical analysis

Basic descriptive statistics (mean and standard deviation for normal data, median and range for non-normal data, and frequency and percentages for categorical data) were computed for all variables. All continuous variables were tested for normality using the Shapiro–Wilk test. Differences in the variables between the sexes were assessed using either two-tailed *t*-test for two means or two proportions, or their respective non-parametric counterparts (Mann–Whitney U test and χ^2^ test) whenever indicated. A separate analysis was performed wherein the differences between the median BMI_K_ and median BMI_T_ values at the bottom and top 10% of the height distribution were assessed using the Mann–Whitney U test; it are in these portions of the height distribution where the two BMI measures are expected to differ the greatest.

The correlations between BMI_K_ and BMI_T_, and between the BMI values and %BF were quantified using Pearson’s correlation or its nonparametric counterpart (Spearman’s correlation) whenever indicated. Agreement between weight classifications based on BMI_K_ and BMI_T_ was determined using Cohen’s κ coefficient.

To assess the utility of BMI_K_ and BMI_T_ in predicting %BF, sex-specific regression models for each BMI type were constructed using polynomial regression analysis. To address possible concerns of violations to normality and homoscedasticity, Huber-White robust standard errors were estimated, with degrees of freedom adjustments for small samples done whenever necessary. Only the linear and quadratic relationships were examined since a cubic relationship between BMI and %BF is not supported by literature. For all linear models, age, WC, smoking history and alcohol intake were used as covariates. This same list of variables plus an added squared BMI term were used as covariates for all quadratic models. The Wald test was then performed to assess goodness-of-fit of the quadratic models by determining if the squared BMI term is statistically important as an explanatory variable. For all constructed regression models, regression diagnostics and procedures for assessment of model adequacy (assessment of linearity and error term independence, model specification, and omitted variables testing) were performed. Because robust polynomial regression analysis corrects issues of heteroscedasticity and violations to normality brought about by outliers or influential observations (through an iterative reweighting process that down-weights the impact of outliers on the coefficient estimates), evaluation for the presence of outliers, leverage and influence as part of model adequacy assessment was no longer necessary. To estimate the relative quality of the models for BMI_T_ and how it performs against the models for BMI_K_, the Akaike information criterion (AIC) and Bayesian information criterion (BIC) were quantified and compared. Lower values of AIC and BIC mean a better fit of the model to the data. For issues of missing data, chained multiple imputation by predictive mean matching was performed.

Since reference values for %BF differ with sex, measures of diagnostic accuracy – sensitivity, specificity, positive predictive values (PPV), negative predictive values (NPV), and likelihood ratios (LR + and LR–) – were determined separately for males and females. Sex-specific receiver operating characteristic (ROC) curves were likewise plotted and the respective areas under the curve (AuROCs) calculated. The optimum BMI_K_ and BMI_T_ cut-off values that distinguish between the normal and overweight–obese weight classifications were identified using the Youden index.

Lastly, post-hoc power analyses were performed at study completion in the event that the target sample size was not achieved in order to assess alignment of the expected effect size with the actual effect size, and to determine whether or not the number of study participants included was sufficient with respect to our study objectives.

Power analyses were performed using R version 4.0.3 (R Core Team, http://www.r-project.org/). All other statistical analyses were carried out using Stata version 15 (StataCorp, College Station, TX). Results were considered statistically significant if *p*-value < 0.05. Ninety-five percent confidence intervals (95% CI) were also calculated for all estimates.

## Data Availability

The datasets generated and/or analysed during the current study are not publicly available due to Philippine Data Privacy Laws in effect but are available from the corresponding author on reasonable request.
